# Utilization of Inexpensive Carbon-Based Substrates as Platforms for Sensing

**DOI:** 10.3390/s18082444

**Published:** 2018-07-27

**Authors:** Minh Tran, Ahmad Fallatah, Alison Whale, Sonal Padalkar

**Affiliations:** 1 Department of Mechanical Engineering, Iowa State University, Ames, IA 50011, USA; mhtran@iastate.edu (M.T.); fallatah@iastate.edu (A.F.); 2 Department of Materials Science and Engineering, Iowa State University, Ames, IA 50011, USA; awhale@iastate.edu; 3 Microelectronics Research Center, Iowa State University, Ames, IA 50011, USA

**Keywords:** gold, electrodeposition, SERS, nanostructures

## Abstract

Gold (Au) has been widely used as a material for Surface Enhanced Raman Spectroscopy (SERS) due to its plasmonic properties, stability and biocompatibility. Conventionally for SERS application, Au is deposited on a rigid substrate such as glass or silicon. The rigid substrates severely limit analyte collection efficiency as well as portability. Here, flexible substrates like carbon cloth and carbon paper were investigated as potential substrate candidates for SERS application. The flexible substrates were coated with Au nanostructures by electrodeposition. Model analyte, Rhodamine 6G was utilized to demonstrate the capabilities of the flexible SERS substrates. Additionally, the pesticide paraoxon was also detected on the flexible SERS substrates as well as on a real sample like the apple fruit.

## 1. Introduction

Detection of trace biological analytes and hazardous chemicals has become increasing important, since serious health risks and environmental problems can be mitigated or prevented by early detection. The field of sensing has demonstrated potential applications in a range of areas including environmental monitoring [1,2], anti-terrorism [3,4], biomedical diagnostics [5], forensic science [6], and food safety [2]. There are several conventional analytical techniques that have been utilized for the ultrasensitive detection of these analytes, some of which include high-performance liquid chromatography (HPLC) and gas chromatography-mass spectroscopy GC/MS [2], capillary electrochromatography (CE) [7], enzyme cycling assays [8], photoluminescence [9], and ion mobility spectrometry [10]. However, these techniques are time-consuming and require expensive equipment. They also require complicated sample pre-treatments, which can be handled only by trained personnel. Thus in order to overcome the limitations of these conventional techniques, researchers have explored and utilized other techniques like Surface Enhanced Raman Spectroscopy (SERS). With the advent of portable Raman Spectroscopy, sensing of trace analytes has become accessible, rapid, sensitive and affordable. Since its discovery in 1974 [11], the field of SERS has grown into an active area of research, including both experimental and theoretical studies [12], and has evolved from its fundamental understanding to promising applications [13].

There are many different types of SERS materials with varying morphologies utilized for detection purposes. Some researchers have used colloidal metallic nanoparticles or their colloidal aggregates in solution for SERS detection [14,15]. Although the preparation of these materials is simple, such approaches do not yield reliable outcomes. The SERS performance has been found to be inconsistent and thus not suitable for non-aqueous applications [16,17]. Other types of SERS materials have rough surfaces [18]. This can be obtained by creating metallic nanoholes [19], concentric rings [20], nanogaps [21], nanoparticles [22,23] and their arrays [24], or nanodisk array [25]. Further, SERS detection was possible by employing porous membrane, [26] latex microspheres, [27] and polystyrene colloidal particles [27]. Additionally, three dimensional (3D) nanostructures such as nanorods, nanocones, etc., have provided precise detection of trace analytes. Although these materials and their varying morphologies showed promising detection capabilities, they are expensive to fabricate and require specialized equipment with trained personnel. In addition, the cost of a conventional SERS substrate like silicon is $0.5/1 g, whereas flexible substrates like paper cost only $0.001/1 g. Moreover, the aforementioned materials and varying morphologies are often fabricated on substrates that are rigid including glass [28,29,30], silicon [31,32], and glass capillary [33]. These underlying rigid substrates are expensive and brittle, which make them ineffective for in-field detection of analytes that can be directly transferred from the target products; for example, in food packaging industry and agricultural fields. 

To improve the usability of materials, with varying morphologies, in SERS detection flexible substrates have been explored including filter paper [16,17,34,35,36,37,38,39], adhesive tape [40], cotton [4,41], carbon cloth [42], polymer nanofibers [43], polymer nanotubes [44], and electrospun poly(vinyl alcohol) nanofibers [35,45]. The fabrication techniques used for such flexible substrates include ink-jet printing [34,39], dip coating [17,35], templating [16], drop casting [40], and electroless deposition [38,42]. Although some of these methods are simple, they require long preparation time or dry time (12–48 h), large material use, special equipment and trained personnel.

In the present investigation, the potential of inexpensive flexible substrates was explored by the fabrication of Au nanostructures on carbon cloth and carbon paper. These Au nanostructures were prepared by electrodeposition. The resulting substrates demonstrated successful detection of different types of organic molecules, including R6G and paraoxon. The detection of analytes using these flexible substrates was reproducible and reliable.

Scanning electron microscopy was used to investigate the morphology, size, density and coverage of Au nanostructures on the underlying flexible substrates. Compositional analysis was done using Energy Dispersive X-ray Spectroscopy. UV-Vis spectroscopy was used for determining optical property of the deposited Au nanostructures. Finally, SERS performance of the flexible substrates was evaluated by Raman spectroscopy.

## 2. Materials and Methods

### 2.1. Materials

The chemicals used for the electrodeposition of Au nanostructures were gold (III) chloride trihydrate (HAuCl_4_·3H_2_O, ≥99.9%) and potassium hydroxide (KOH, ≥85.8%). These chemicals were purchased from Sigma-Aldrich (St. Louis, MO, USA) and Fisher Scientific (Hanover park, IL, USA), respectively. The substrates used for electrodeposition of the Au nanostructures were carbon cloth (AvCarb Material Solutions, 1071 HCB) and wet-proofed carbon paper (Toray, 060) purchased from Fuel Cell Store (College Station, TX, USA). The chemicals used for cleaning the substrates were acetone, hydrochloric acid (HCl, 36.5–38.0%), and nitric acid (HNO_3_, 68.0–70.0%), purchased from Fisher Scientific. For SERS experiments, Rhodamine 6G dye (R6G, 99%) and paraoxon-ethyl (C_10_H_14_NO_6_P, ≥90%) were purchased from Sigma-Aldrich. Deionized (DI) water was used for preparing all precursor and analyte solutions.

### 2.2. Fabrication of Au Nanostructures via Electrodeposition

**Figure 1:**
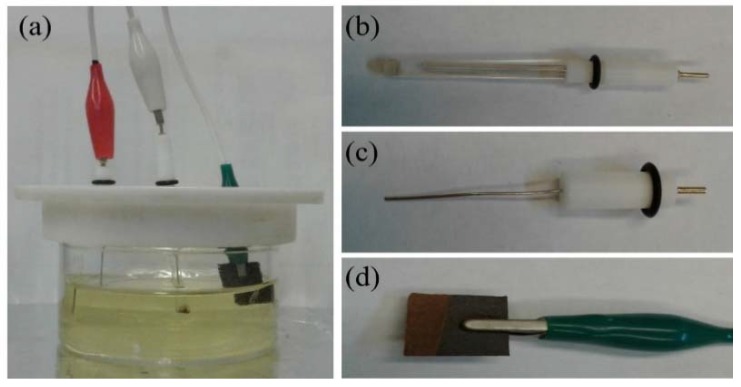
Photographs of the electrochemical cell (**a**), which consists of an Ag/AgCl reference electrode (**b**), platinum counter electrode (**c**) and carbon paper as working electrode (**d**).

The Au nanostructures were fabricated via electrodeposition using a three electrode electrochemical cell as shown in Figure 1. The electrochemical cell consisted of an Ag/AgCl reference electrode (Figure 1b), a 2 mm diameter platinum wire counter electrode (Figure 1c) and carbon cloth or carbon paper, as the working electrode (Figure 1d). Prior to electrodeposition, the carbon paper was cleaned by ultrasonicating in an acetone bath for 10 min. This was followed by cleaning with hydrochloric acid and nitric acid for 1 min each. For carbon cloth, the cleaning procedure was carried out in three steps. The carbon cloth was first immersed in acetone for 1 h, followed by submerging in boiling DI water for 10 min. In the final step, the carbon cloth was cleaned using an acetone bath in an ultrasonicator for 10 min [46]. The substrates were rinsed with DI water after each cleaning step.

For the electrodeposition of Au nanostructures, the electrolyte solution was prepared by dissolving HAuCl_4_ in DI water to obtain a concentration of 3 mM. The pH of the electrolyte was adjusted to 3 using KOH. The electrodeposition was carried out at room temperature for 70 min, at an applied potential of −0.8 V [47].

To prepare the dye solution, 0.8 mg of R6G was dissolved in 5 mL of DI water, and was utilized as a stock solution. Similarly, paraoxon stock solution was prepared by mixing 30 μL of oily concentrated paraoxon with 1 mL DI water. To prepare samples for SERS experiments, a small volume of the stock solution was diluted to the desired concentration. Further, 300 μL of the diluted solution was drop cast onto the Au nanostructures fabricated on either carbon cloth or carbon paper. The substrates were then dried under ambient conditions. Prior to drop casting, the wettability of the substrates was improved by oxygen plasma treatment, for 1 min under medium radio frequency power level (11 W) by a plasma cleaner (PDC-001, Harrick Plasma, Ithaca, NY, USA).

### 2.3. Characterization

The Au nanostructure morphology was studied using scanning electron microscopy (SEM) using a FEI Quanta-250 SEM instrument operating at 10 kV accelerating voltage. The SEM instrument was equipped with an Oxford Aztec Energy Dispersive X-ray (EDX) analysis system, which was used to conduct compositional analyses on the sample surface. The optical properties were studied using UV-Vis absorption spectroscopy, which used a Perkin Elmer Lambda 25 spectrophotometer to obtain the necessary data. To prepare samples for UV-Vis measurements, each sample was immersed in 1 mL of DI water and sonicated at the highest power for 5 min to detach the Au nanostructures from the substrates. The SERS measurements were performed at room temperature on a Renishaw Dispersive Raman Spectrometer with Ar-ion laser at 488 nm, using 50× objective lens, with incident power of 5 mW for 4 accumulations. Here, each accumulation was of 30 s duration. The SERS spectra were collected from several samples and from random regions on each sample to confirm reproducibility and uniformity respectively.

## 3. Results and Discussion

### 3.1. Characterization of Gold Nanostructures

**Figure 2:**
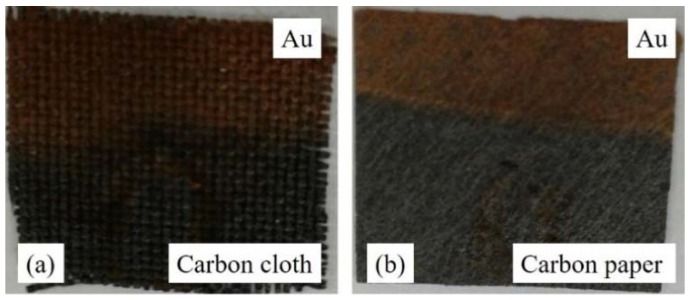
Photographs showing carbon cloth (**a**) and carbon paper (**b**) after electrodeposition of Au nanostructures.

The digital photographs of the as-prepared carbon cloth and carbon paper electrodeposited with Au nanostructures are shown in Figure 2. The photographs clearly show a uniform and continuous deposition of Au; a dark golden color on both carbon cloth and carbon paper. The detailed electrodeposition conditions followed in this work were reported elsewhere by our group [47]. 

**Figure 3:**
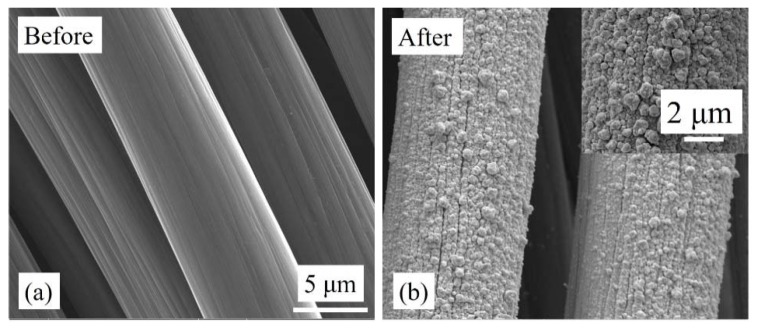
SEM images of carbon cloth (**a**) before and (**b**) after electrodeposition of Au nanostructures. The inset shows Au-electrodeposited carbon cloth at higher magnification.

**Figure 4:**
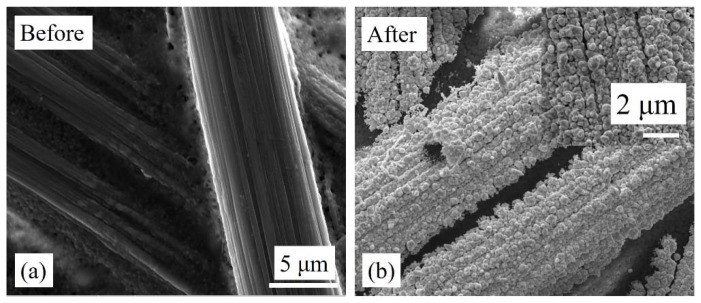
SEM images of carbon paper (**a**) before and (**b**) after electrodeposition of Au nanostructures. The inset shows Au-electrodeposited carbon paper at higher magnification.

Figure 3 and Figure 4 show carbon cloth and carbon paper before and after Au nanostructure deposition. Prior to deposition, the carbon cloth and carbon paper have a smooth surface with uniform width of its constituent strands. After the deposition of Au, the thickness of the constituent strands increased and was clearly seen in the SEM images (Figure 3b and Figure 4b). A compact layer of Au nanostructures was observed, which covered the surface of the substrates. The surface of the deposited Au layer appeared to be rough as a result of the coalescence of Au nanostructures. Above the compact layer, a sub-monolayer of Au nanostructures was also observed (insets of Figure 3b and Figure 4b). The details of the deposition mechanism have been explained in our previous work [47,48].

**Figure 5:**
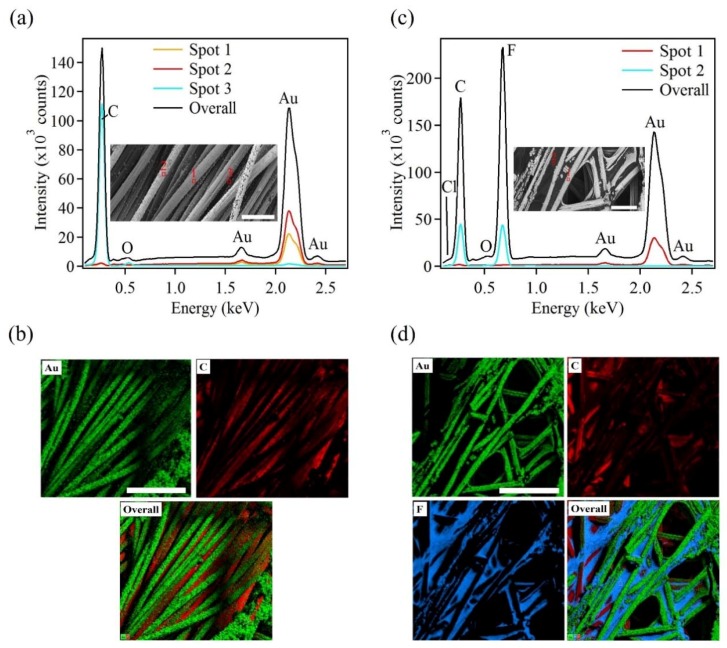
Energy dispersive x-ray (EDX) spectra and mapping analysis of Au nanostructures electrodeposited on (**a**,**b**) carbon cloth, and (**c**,**d**) carbon paper, respectively. The insets show the different regions where EDX patterns were taken. The scale bars in the insets and the EDX mapping are 50 μm and 100 μm, respectively.

The chemical composition of the as-synthesized samples was determined by EDX. The EDX spectra obtained from the carbon cloth and carbon paper samples are presented in Figure 5. From the carbon cloth, the EDX spectra indicated a strong presence of Au. The spectra were recorded from different regions with varying depths on the sample as indicated in the inset of Figure 5a. It can be seen that the intensity of the Au signal from different regions varied. This was due to the variation in the deposition of Au with respect to the depth of the constituent strands that make up the carbon cloth. From the SEM images it was clear that the Au deposition occurred on the uppermost strands whereas the strands underneath remained partially or fully uncoated. Along with the Au, the EDX spectra showed the presence of C and trace amount of O. The presence of C was due to the underlying carbon cloth substrate. The trace amount of O was attributed to small amount of surface contamination. Figure 5b shows the elemental mapping obtained from the Au coated carbon cloth. The elemental maps indicate the presence of Au and C.

Similarly, compositional analysis on the Au coated carbon paper sample was performed. Figure 5c presents EDX spectra taken at different regions of varying depths on the sample as indicated by the inset in Figure 5c. Here, EDX spectra indicated similar trends, when compared to the carbon cloth substrate. The EDX spectrum, taken from the topmost layer of the sample, showed high intensity peaks of Au, F and C. The next spectrum exhibited lower intensity peaks, which was taken from a deeper portion of the sample. The presence of Au and C was clearly seen from the EDX data. The presence of Cl and F ions were attributed to the remnant Au precursor and adhesive resin, used in the preparation of the carbon paper, respectively [46]. The indication of O in the spectra was due to surface contamination. From the EDX data, it was inferred that the Au deposition varied along the depth of the sample. Thus future efforts will be focused on obtaining a more uniform and complete coating on layered substrates such as carbon cloth and carbon paper. Table 1 shows mass fraction analyses of the chemical elements obtained by EDX. Here, the table indicates that Au was the primary element deposited on the carbon-based substrates.

**Figure 6:**
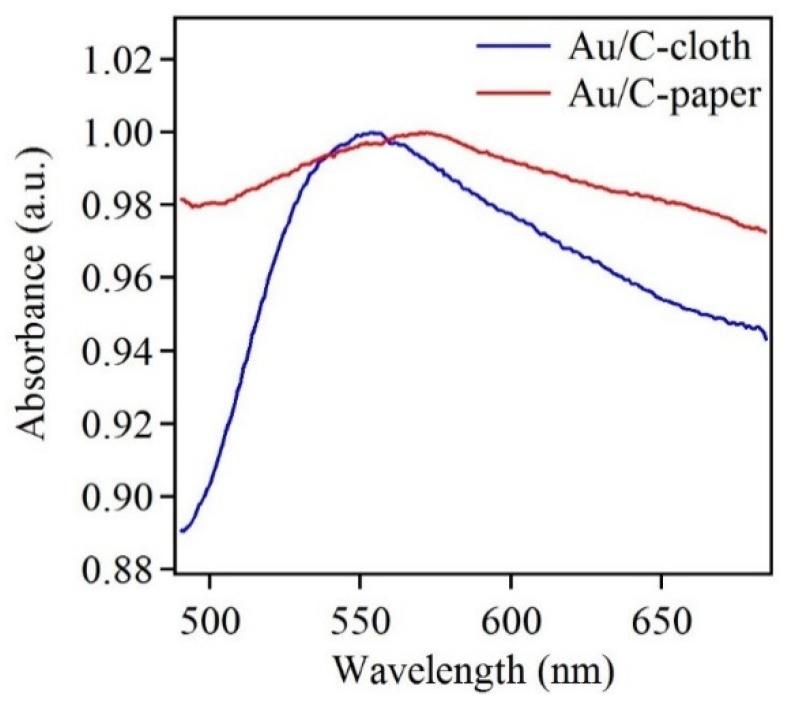
Normalized UV-Vis spectra of Au-electrodeposited carbon cloth and carbon paper.

Along with the EDX data, UV-Vis absorption data was also obtained. The absorption spectra from samples of Au electrodeposited on carbon cloth and carbon paper are presented in Figure 6. Both spectra show characteristic absorption peaks of Au. The red-shift beyond 550 nm indicates that the Au nanostructures were large in size. Additionally, a large full width half maximum of the absorption spectra indicates a broad size distribution [49]. Thus, the UV-Vis data conformed the SEM data, which implies large nanostructure size and broad size distribution.

### 3.2. Electrodeposited Gold Nanostructures for SERS Application

**Figure 7:**
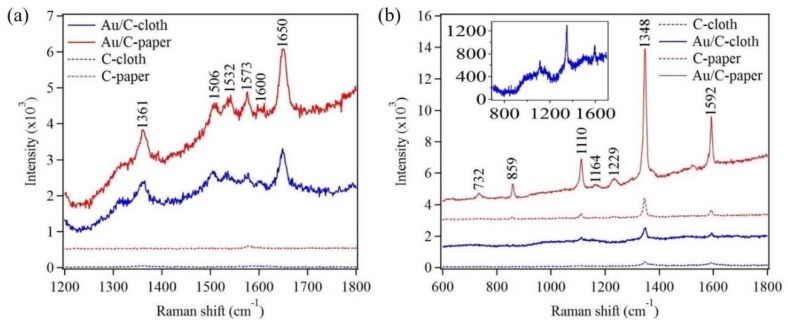
Raman spectra of 10^−5^ M and 10^−6^ M R6G drop casted on Au coated carbon cloth and carbon paper respectively (**a**). Raman spectra of 10^−2^ M paraoxon drop casted on both flexible substrates in the presence and absence of Au nanostructures (**b**).

The fabricated Au nanostructures were evaluated for the detection of R6G and paraoxon via SERS. The organic molecule, R6G, has been widely used for SERS studies since it has well documented and clearly defined Raman modes. Figure 7a shows Raman spectra of R6G (10^−5^ M), on flexible substrates like carbon cloth and carbon paper, in the presence and absence of Au nanostructures. The sample preparation was carried out by drop casting R6G on the flexible substrates. The Raman modes of R6G were not observed on substrates in the absence of Au nanostructures. However, in the presence of Au nanostructures characteristic Raman modes of R6G were clearly observed on both carbon cloth and carbon paper (Figure 7a and Table 2). 

In order to further test the sample’s capabilities to detect real and small organic molecules, an organophosphorus pesticide, paraoxon was used. Figure 7b shows Raman spectra of paraoxon (10^−2^ M), which was drop casted on flexible substrates in the presence and absence of Au nanostructures. A very weak Raman signal of paraoxon was observed on the flexible substrates in the absence of Au nanostructures. However, strong characteristic Raman modes for paraoxon were observed, on flexible substrates, in the presence of Au nanostructures (Figure 7b and Table 2). The inset in Figure 7b clearly shows the Raman mode of paraoxon on Au nanostructures electrodeposited on carbon cloth [50]. The increase in the Raman signal was attributed to the localized electromagnetic field enhancement due to the presence of plasmonic nanostructures. The electrodeposited Au nanostructures not only increase the surface area but create small crevices, which are conducive to trapping of photons for a finite amount of time. This trapping of photons leads to an increase in the electromagnetic field in the small crevices, thus generating a clear and intense Raman signal [51,52].

Additionally, it is noteworthy that the Raman signal intensity, in all samples, was always higher for the carbon paper compared to carbon cloth. This increase in signal intensity was attributed to the different surface wetting properties of both flexible substrates. The carbon paper was observed to be more hydrophilic compared to the carbon cloth, which governed the absorbing capability of the probe molecules.

### 3.3. In-Field Testing Using Gold Nanostructures for Detection of Paraoxon via SERS

**Figure 8:**
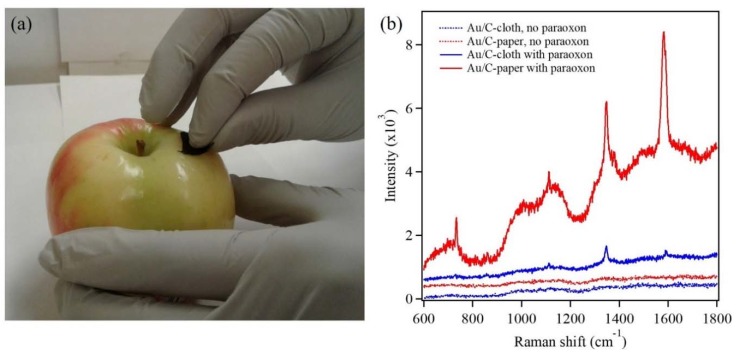
Wiping the contaminated area on an apple’s skin by carbon cloth electrodeposited with Au nanostructures for detection of paraoxon (10^−2^ M) via SERS (**a**). Raman spectra showing characteristic Raman modes of paraoxon (**b**).

The in-field testing was mainly focused on the viability of Au nanostructures on flexible substrates to detect analytes, which are present on food products. The analyte in the present investigation was paraoxon (10^−2^ M), which was drop casted on the skin of an apple and was dried under ambient conditions. Further, the paraoxon was directly transferred onto the Au nanostructures electrodeposited on both the flexible substrates. This was carried out by immersing the flexible substrate, with Au nanostructures, in DI water and was used to wipe the paraoxon-infected area on the apple (Figure 8a). These flexible substrates were then used for the detection of paraoxon via SERS. Figure 8b shows Raman spectra of the flexible substrates before and after paraoxon transfer. The characteristic Raman modes of paraoxon were observed for both flexible substrates. Thus, it can be clearly seen that paraoxon was successfully transferred to the substrates and was also successfully detected. Thus, this investigation validates the stability and robustness of the Au nanostructure coated flexible substrates. It further demonstrates that a flexible substrate is more valuable than a rigid SERS substrate in such in-field testing applications. These flexible SERS substrates can find potential applications in not only the food packaging industry but also in agricultural fields, thus empowering farmers to regulate the pesticide usage in their fields. The present investigation has worked with concentration of paraoxon being on the higher side. However, this is a proof of concept study to demonstrate the viability of a flexible substrate as a SERS substrate and its potential application. 

## 4. Conclusions

In summary, Au nanostructures were successfully electrodeposited on flexible substrates including carbon cloth and carbon paper. The flexible substrates were thoroughly characterized by SEM, EDX, and UV-Vis to indicate the presence of Au nanostructures. These flexible substrates with Au nanostructures were then used to demonstrate their viability as SERS substrates for the detection of R6G and paraoxon. Additionally, successful in-field testing of these flexible substrates was carried out to detect paraoxon on an apple’s skin. The SERS data indicated characteristic Raman modes for all analytes that were investigated. The detection of analytes was possible due to the plasmonic properties of the Au nanostructures, which led to the localized enhancement of the electromagnetic field. 

## Figures and Tables

**Table 1:** Mass fraction analysis of chemical elements obtained by EDX.

Element	C	O	F	Cl	Au	Total
**Au/C-Cloth**	2.86	0.20	N/A	0.03	96.91	100.00
**Au/C-paper**	2.56	0.37	0.37	0.07	96.62	100.00

**Table 2:** Raman mode assignments for R6G and paraoxon pesticide corresponding to Figure 7.

Raman Mode (cm^−1^)	Assignment for R6G	Reference
1361	Aromatic C–C stretching, in-plane C–H bending	[53,54,55,56]
1506, 1532	Aromatic C–C stretching, C–N stretching, C–H bending, N–H bending	[53,54,55]
1573, 1600	Aromatic C–C stretching, in-plane N–H bending	[53,54,55]
1650	Aromatic C–C stretching, in-plane C–H bending	[53,54,55,56]
**Raman Peak (cm^−1^)**	**Assignment for Paraoxon**	**Reference**
732	NO_2_ scissor, C–C bending	[3,57]
859	NO_2_ scissor (Aromatic–NO_2_)	[3,57]
1110	C–H band (in plane)/NO_2_ asymmetric stretching	[3,57]
1348	Symmetry stretching NO_2_	[3,57]
1592	Phenyl ring vibration	[3,57]

## Supplementary Materials

Supplementary File 1
