# Supplementary material for: Utilization of Inexpensive Carbon-Based Substrates as Platforms for Sensing
Source: Sensors (Basel). 2018 Jul 27;18(8):2444. doi: 10.3390/s18082444 (PMC6111970; doi:10.3390/s18082444)
Supplement: Supplementary File 1 [file sensors-18-02444-s001.pdf]

sensors

MDPI

Supporting Information

Utilization of Inexpensive Carbon-Based Substrates as Platforms for Sensing

Minh Tran [1], Ahmad Fallatah [1], Alison Whale [2] and Sonal Padalkar [1,3,*]

[1] Department of Mechanical Engineering, Iowa State University, Ames, IA 50011, USA; mhtran@iastate.edu (M.T.); fallatah@iastate.edu (A.F.)
[2] Department of Materials Science and Engineering, Iowa State University, Ames, IA 50011, USA; awhale@iastate.edu
[3] Microelectronics Research Center, Iowa State University, Ames, IA 50011, USA
* Correspondence: padalkar@iastate.edu; Tel.: +1-51529-46066

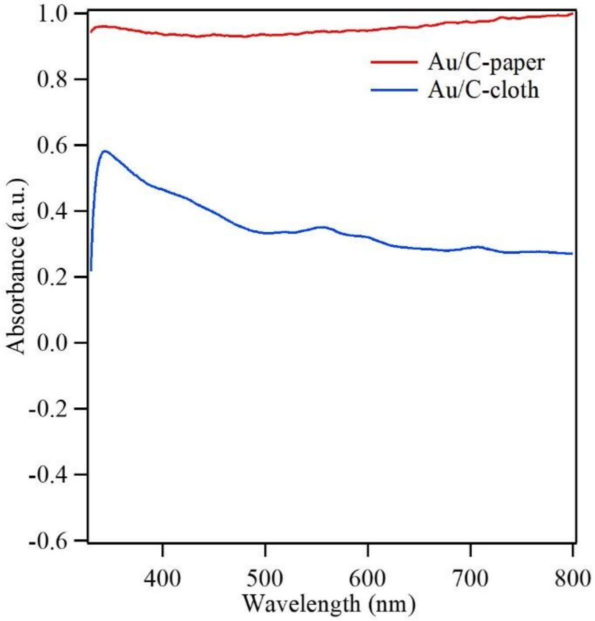

Figure S1. UV-Vis spectra of bare carbon cloth and carbon paper.

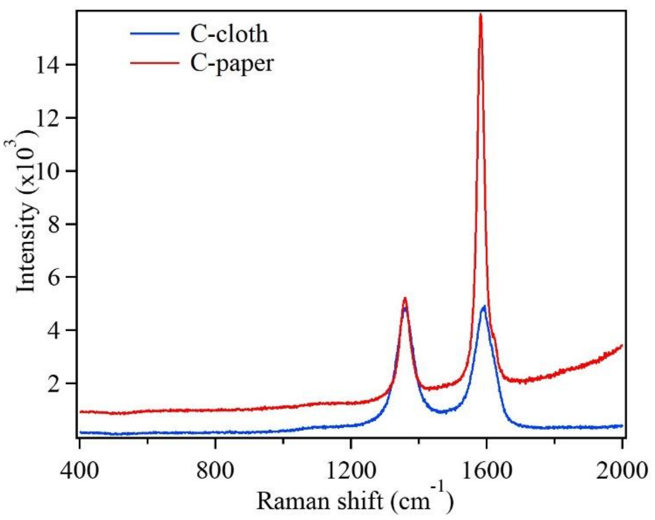

Figure S2. Raman spectra of bare carbon cloth and carbon paper.

Here, it is important to note that the Raman modes of the bare carbon based substrates do not coincide entirely with the Raman modes of the analyte. Thus successful detection of the analyte was possible.
